# False Positive F-18 FDG PET/CT of Skeletal Metastasis Due to Solitary Eosinophilic Granuloma

**DOI:** 10.4274/Mirt.296

**Published:** 2013-12-10

**Authors:** Robert Mansberg, Bao Ho, Chuong Bui, Cathie Crombie

**Affiliations:** 1 Nepean Hospital, Nuclear Medicine and PET, Penrith, Australia; 2 Nepean Hospital, Oncology Department, Penrith, Australia

**Keywords:** Positron Emission Tomography /computed tomography, Fluorodeoxyglucose F18, Eosinophilic granuloma, image-guided surgery

## Abstract

A 31 year old female with a 3 month history of focal right mid posterior thoracic pain, and solitary lytic lesion in the right 7th rib posteriorly on bone scan (SPECT/CT) was referred for PET/CT to identify alternate site for biopsy in suspected malignancy. The patient had no significant past medical history and was afebrile with mildly elevated inflammatory markers. A solitary intensely FDG avid focus was demonstrated localised to a well-defined lytic lesion with partial cortical erosion on the posterior aspect of the right 7th rib. No adjacent soft tissue abnormality was seen. No other site of biopsy was demonstrated. As malignancy (metastatic or primary) was not excluded, CT guided localisation with hookwire and blue dye injection was performed immediately prior to partial resection of the right 7th rib. Histopathology confirmed the diagnosis of eosinophilic granuloma.

**Conflict of interest:**None declared.

## INTRODUCTION

Eosinophilic Granuloma (Histiocytosis X) is the benign form of the clinical variants of Langerhans cell histiocytosis and characterized by single or multiple skeletal lesions predominantly in children, adolescents and young adults 1. We report a 31 year old woman who presented to her local doctor with severe pain in the right posterior thoracic region. A solitary lytic lesion was demonstrated in the right 7th rib posteriorly on bone scan (SPECT/CT) and subsequently on PET/CT. As no alternative site for biopsy was revealed accurate localisation of the lesion with CT guided hookwire positioning and blue dye injection was performed prior to excision biopsy. Histopathology confirmed the lesion to be due to solitary eosinophilic granuloma.

## CASE REPORT

**Figure 1:**
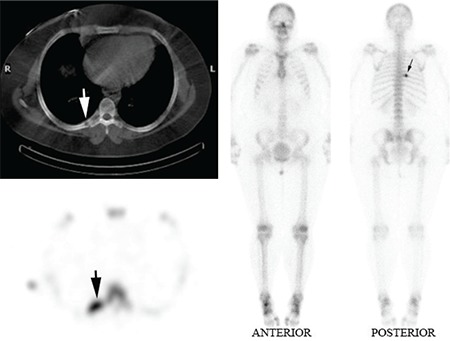
A solitary focus of intense osteoblastic activity demonstrated inthe right 7th rib posteriorly (Solid black arrow) on planar and SPECT imagesand (Solid white arrow) on axial CT slice

A 31 year old female presented with a three month history of right mid posterior thoracic pain. A bone scan with SPECT/CT was performed following the administration of 750 MBq Technetium 99m MDP on a GE Hawkeye Gamma Camera. A solitary focus of intense osteoblastic activity was demonstrated in the right 7th rib posteriorly (Solid black arrow ) on planar and SPECT (Solid white arrow) on axial CT slice (Figure 1).

**Figure 2:**
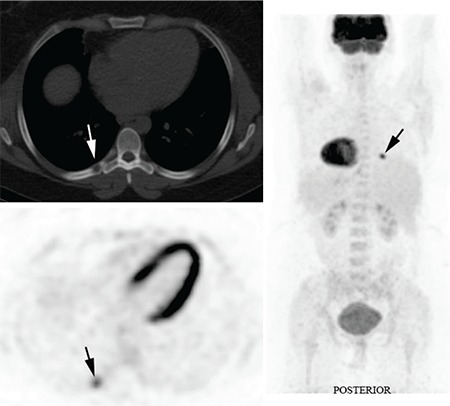
The PET study revealed a solitary focal intense FDG uptake inthe right 7th rib posteriorly (solid black arrow). The concurrent low dose CTscan of the Philips Gemini 64 PET/CT camera confirmed the presence of awell-defined lytic lesion with partial bony erosion at the site of the intenselyincreased FDG uptake (solid white arrow)

The patient was referred for a FDG PET/CT study to assess for alternate sites for tissue biopsy in suspected malignancy. A FDG PET study and low dose CT study was performed following administration of 240 MBq F18 FDG on a Philips Gemini 64 PET/CT camera. The PET study revealed a solitary focal intense FDG uptake in the right 7th rib posteriorly (solid black arrow). The concurrent low dose CT scan of the Philips Gemini 64 PET/CT camera confirmed the presence of a well-defined lytic lesion with partial bony erosion at the site of the intensely increased FDG uptake (solid white arrow) (Figure 2).

**Figure 3:**
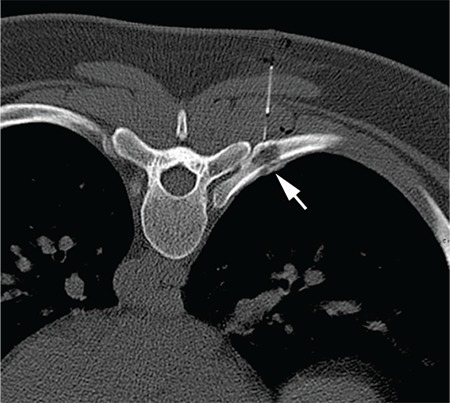
CT guided hookwire localization and injection of blue dye priorto an excision biopsy of the lesion (solid white arrow)

In the absence of an alternate site for biopsy the patient underwent CT guided hookwire localization and injection of blue dye prior to an excision biopsy of the lesion (solid white arrow Figure 3). The gross specimen of the resected right 7th rib lesion revealed a friable tan tissue with flecks of yellow. Microscopically an infiltrate of histiocytes, sheets of Langerhan’s cells and inflammatory cells were present, confirming the diagnosis of eosinophilic granuloma.

## LITERATURE REVIEW AND DISCUSSION

Eosinophilic granuloma is a benign form of the three clinical variants of Langerhans cell histiocytosis. It is characterized by single or multiple skeletal or lung lesions, and it predominantly affects children, adolescents, and young adults. Solitary lesions are more common than multiple lesions. There is a two to one male preponderance.

In skeletal involvement, eosinophilic granuloma can be asymptomatic or present with local pain, swelling and tenderness and ESR may be elevated. The lesion may cause endosteal scalloping or a periosteal reaction (1).

PET with F18-FDG is recommended for the primary diagnosis and staging as well as for the detection of recurrences in patients with different types of tumor. However, conventional radiography is still the method of choice for the initial diagnosis of a primary bone tumor and the differentiation between malignant and benign intraosseous lesions. In many cases, CT and MRI are needed to assess the nature and the morphology of a suspicious lesion. However, the sensitivity and specificity of CT and MRI may be highly variable. Bone scan is highly sensitive but is also not specific (2,3,4).

The sensitivity reported in the literature for PET scanner exceeds 90%, whereas the specificity also remains lower and highly variable, ranging from 65% to 80% (2,5,8). Low-grade tumors, which frequently show a low F18-FDG uptake, provide the main reason for false-negative results. False-positive results may be caused not only by acute inflammatory lesions but also by some benign diseases with an inflammatory component, such as eosinophilic granuloma, fibrous dysplasia, or aneurysmal cysts (6,8,9,10,11).

This case highlights eosinophilic granuloma as a potential interpretative pitfall demonstrating that FDG uptake is not specific for malignancy. Many different physiological variants and benign pathological conditions can also exhibit increased glucose metabolism. Such false-positive FDG uptake often arises outside the area of primary interest and may mimic malignant disease, thereby confounding accurate interpretation of PET/CT studies.
